# How to manage coronary sinus venous perforation during left ventricular lead implantation: a case report

**DOI:** 10.1093/ehjcr/ytaf241

**Published:** 2025-06-05

**Authors:** Anoop K Gupta, Jyotika Gupta, Siddhant Jain, Pooja Shah

**Affiliations:** Department of Cardiology, Epic Hospital, Room Number 6, OPD Block, Rajpath Rangoli Road, Ahmedabad, Gujarat 380052, India; Life Sciences, Ahmedabad University, Ahmedabad, Gujarat 380009, India; Shalby Hospital, Janjeerwala Square, Indore, Madhya Pradesh 452003, India; Epic Hospital, Ahmedabad, Gujarat 380052, India

**Keywords:** Case report: Cardiac resynchronization therapy, Coronary sinus perforation, Glue, Quadripolar LV lead

## Abstract

**Background:**

Coronary sinus venous branch stenosis is an uncommon entity. Balloon dilatation of venous tributary may not always be safe. We report a case of venous perforation following balloon dilatation, which was managed by glue occlusion and completion of cardiac resynchronization therapy (CRT) implantation.

**Case summary:**

A 50-year-old man was diagnosed with non-ischaemic cardiomyopathy with New York Heart Association (NYHA) Class III dyspnoea despite optimal medical therapy. The electrocardiogram showed a left bundle branch block with a QRS duration of 168 ms, and 2D echocardiography revealed dilated cardiomyopathy with a left ventricular ejection fraction of 20%. The patient was taken for CRT implantation; however, there was severe stenosis in the posterolateral vein noted during left ventricular (LV) lead implantation, hindering LV lead advancement. Following balloon dilatation, there was perforation of the vein with hypotension. The perforation was sealed with glue injection (*n*-butyl-2-cyanoacrylate), and LV lead placement was performed. At the 4-year follow-up, the patient is in NYHA Class I and the ejection fraction improved to 60%, with an excellent LV threshold and good synchronization.

**Discussion:**

Glue (*n*-butyl-2-cyanoacrylate) occlusion can manage coronary sinus perforation with suitable long-term LV lead parameters.

**Learning points**

- Coronary sinus target vein stenosis can be managed by balloon dilatation; however, it carries the remote risk of perforation.
- Venous perforation following balloon dilatation can be managed by glue occlusion and completion of the procedure without lead malfunction.

## Introduction

Cardiac resynchronization therapy (CRT) is a well-established treatment for drug-refractory heart failure patients with electrical dyssynchrony (underlying left bundle branch block (LBBB)); the implant technique has become relatively easy over the period with good hardware.^1^ Challenging anatomy and small and tortuous veins may hamper the left ventricular (LV) lead placement in targeted veins, affecting the procedure's success and long-term result.^2^

Coronary sinus venous stenosis can be encountered in 7%–10% of patients. Various tools and techniques have been described to overcome venous stenosis and achieve optimum LV lead implantation.^3,4^ We report a posterolateral vein perforation following balloon dilatation, which was managed by glue occlusion and completion of CRT implantation.

## Summary figure

**Figure:**
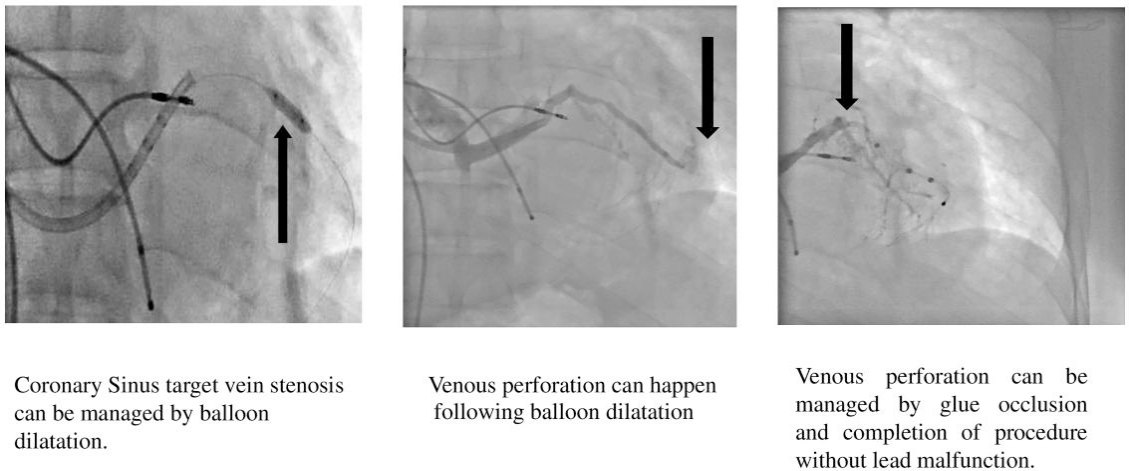


## Case report

**Figure 1:**
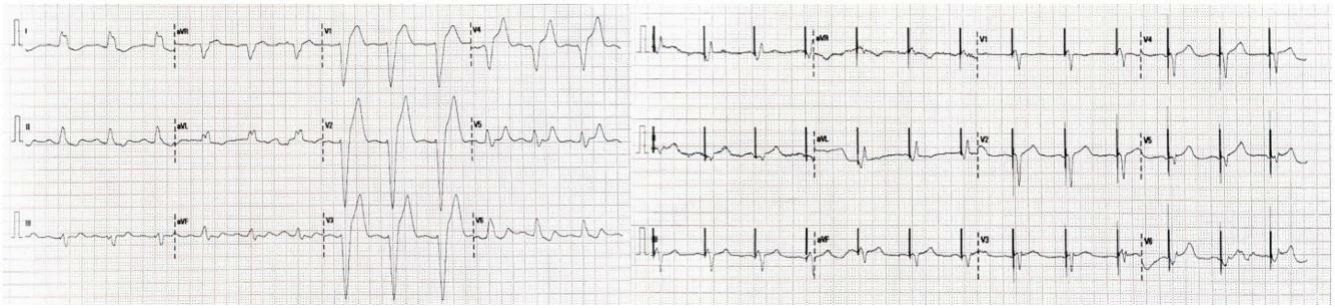
Twelve lead electrocardiogram before and after cardiac resynchronization therapy implantation. The left panel shows a baseline electrocardiogram with a typical left bundle branch block, and the right panel shows ‘A’ Sense and ‘Biventricular’ pacing.

A 50-year-old man with non-ischaemic cardiomyopathy presented with NYHA Class III dyspnoea; he was on optimal heart failure medications, including angiotensin receptor/neprilysin inhibitor (ARNI), beta-blocker, mineralocorticoid receptor blocker (MRA), and sodium-glucose co-transporter 2 (SGLT-2) for 1 year. On admission, the patient’s pulse rate is 90 b.p.m., blood pressure is 90/60 mmHg, and oxygen saturation is 94% on room air, respectively. Clinical examination revealed soft S1 and 2/6 PSM in the mitral area; there was mild basal crepitation. An electrocardiogram (ECG) showed a LBBB with a QRS duration of 168 ms (*Figure 1*, left panel). 2D Echocardiography revealed dilated LV with global hypokinesia, moderate mitral regurgitation, and mild pulmonary hypertension. The left ventricle internal diameter in diastole [LVID d)] was 64 mm and the left ventricle end diastolic volume (LVEDV) was 220 mL, with a LV ejection fraction (LVEF) of 20%. His coronary angiography was normal with no significant stenosis, and cardiac magnetic resonance imaging (MRI) was suggestive of idiopathic dilated cardiomyopathy with no late gadolinium enhancement (LGE) uptake.

The patient was taken up for cardiac resynchronization therapy-pacing (CRT-P) implantation, venogram guided three separate left axillary venous accesses. The right ventricular (RV) lead (St. Jude Medical—Tendril STS 2088—58 cm) was conventionally implanted at mid-septum with preformed stylet, and the right atrial (RA) lead was implanted at the RA appendage (St. Jude Medical—Tendril STS 2088—52 cm).

**Figure 2:**
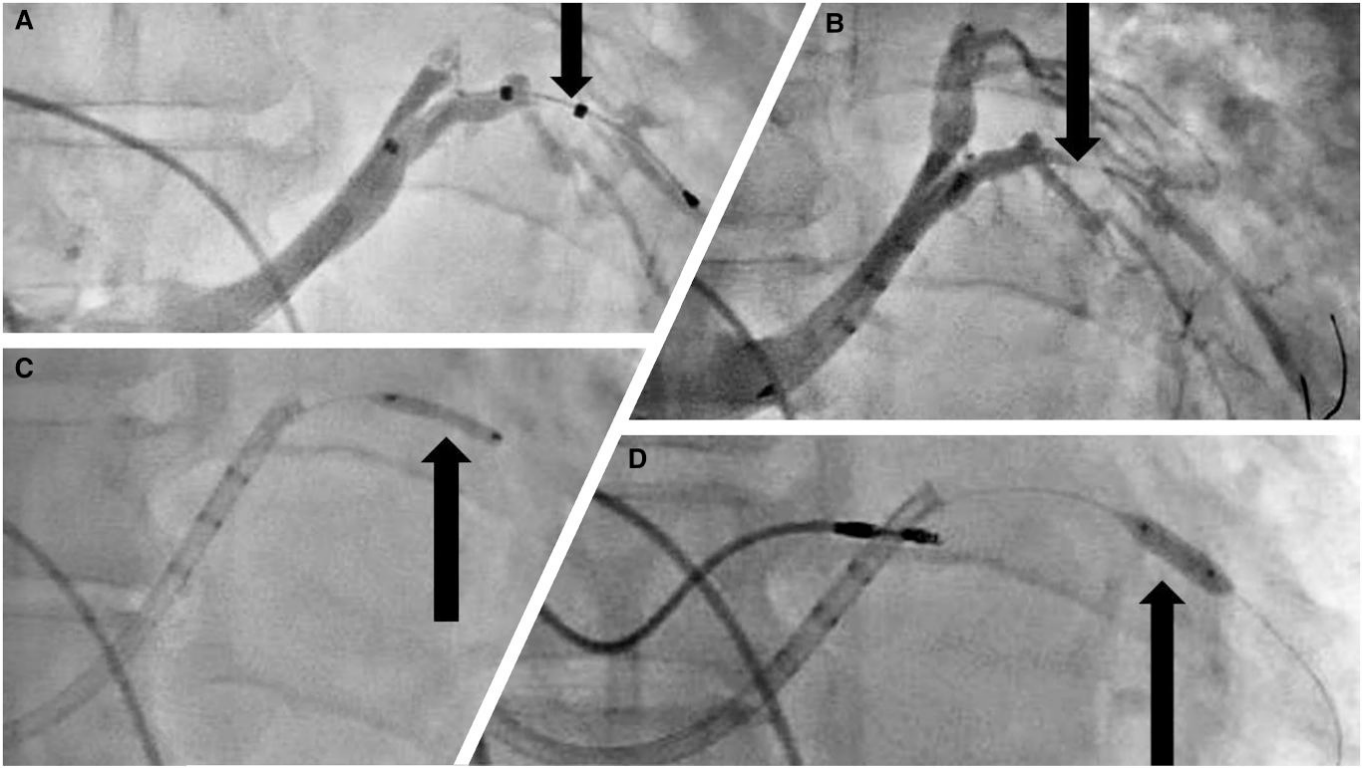
Coronary sinus venogram shows (*A*) quadripolar left ventricular lead (arrow) cannot cross the stenosis in the proximal part of the posterolateral vein. (*B*) Severe proximal stenosis (arrow) of the posterolateral vein. (*C*) Balloon dilatation with 2.0 × 15 mm non-compliant Quantum apex balloon (arrow). (*D*) Balloon dilatation with 3.0 × 12 mm non-compliant Quantum apex balloon (arrow).

A coronary sinus venogram was performed after cannulation. The distal part of the posterolateral vein was found to be the most optimal/preferred, but its proximal part was significantly stenosed. The posterolateral vein was selectively cannulated, and wiring of the lateral branch was performed using a 0.014 Runthrough floppy wire. The quadripolar LV lead (Quartet 1458Q—86 cm) was advanced over the wire; however, due to severe stenosis, it could not be advanced beyond the stenosis (*Figure 2A* and *B*).

The lesion was crossed with a 2.0 × 15 mm non-compliant (NC) coronary balloon, Quantum apex; Boston Scientific (*Figure 2C*). The lesion was dilated, and lead was advanced along the percutaneous transcatheter coronary angioplasty (PTCA) wire. However, it did not work. The sub-select catheter was advanced over the inflated balloon (anchor balloon technique) to cross the lesion, but failed to advance. Hence, the decision was taken to dilate the lesion with a more enormous balloon.

**Figure 3:**
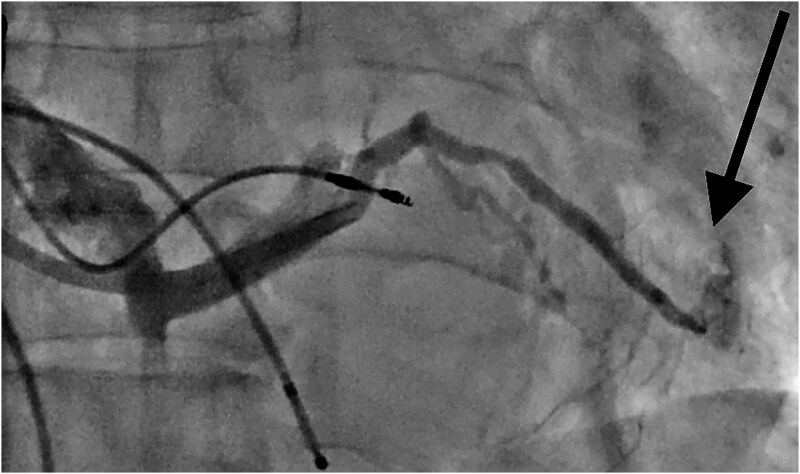
Extravagation of dye (arrows) due to venous perforation after balloon dilatation.

The Runthrough wire was exchanged with Grand Slam PTCA wire for extra support. The lesion was dilated with a 3.0 × 12 mm NC Quantum apex balloon; Boston Scientific at 12 atmospheric pressures (*Figure 2D*); following balloon dilatation, the balloon tracking to the distal part of the vein was smooth. The patient developed hypotension with chest pain; a check venogram revealed venous perforation with dye in the pericardium (*Figure 3*). Echocardiography showed mild pericardial effusion. Intravenous fluid and inotropes were started, and preparation was made for pericardiocentesis. The patient was observed for haemodynamics, and repeat echocardiography did not reveal any increase in pericardial fluid; hence, the LV lead was tracked over the Runthrough PTCA wire and placed at the distal part of the posterolateral vein.

**Figure 4:**
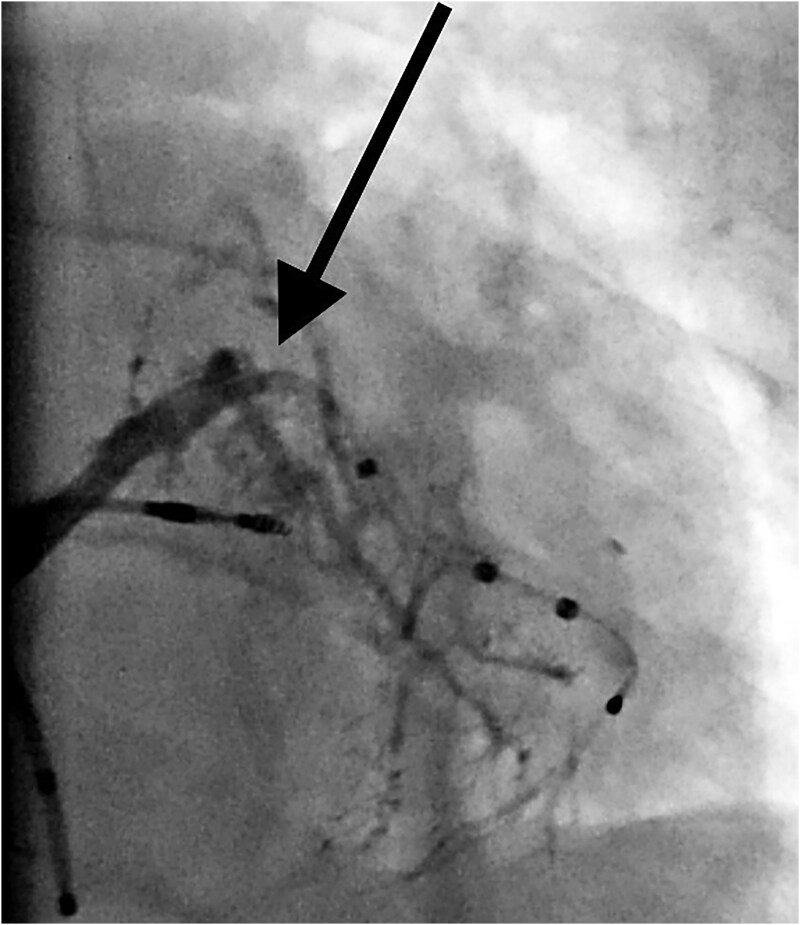
Glue injection through microcatheter (arrow) in posterolateral vein after left ventricular placement shows obliteration of vein (no dye seen inside and outside the vein).

Given an active leak from a posterolateral vein, it was decided to close the venous perforation with local glue injection (*n*-butyl-2-cyanoacrylate). The microcatheter was advanced over the PTCA wire and placed just before the perforation site, and 0.5 mL glue and lipiodol solution were injected through the microcatheter (*Figure 4*).

**Figure 5:**
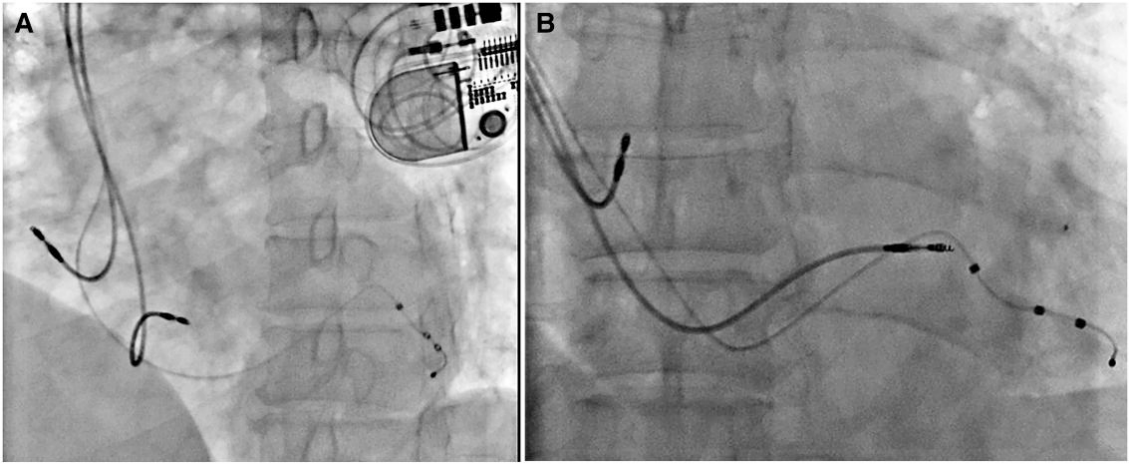
Final position of leads in fluoroscopy (*A*) left anterior oblique (LAO) 40-degree view and (*B*) antero-posterior (AP) view.

The LV lead parameters were satisfactory after glue injection with R wave 16 mV, impedance 722 Ω, and threshold 1.0 V. The haemodynamics improved after resynchronization and inotropes were stopped. All electrodes were connected to a biventricular pulse generator (St. Jude Medical—Allure Quadra PM 3542) implanted subcutaneously in the pocket (*Figure 5A* and *B*). Post-implantation ECG showed a QRS duration of 100 ms (*Figure 1*, right panel) and device parameters were good.

Follow-up of the patient after 1 and 6 months showed that the patient had NYHA Class II, with EF improved to 40%, and all implanted leads were in the same place with good pacing and sensing parameters. The lead parameters are good at 4 years follow-up, and the patient is in NYHA Class 1, with LVEF of 60%.

## Discussion

Many factors have been described to determine the CRT response, including the ability to implant the LV lead at the desired location. Sometimes, there is still some difficulty in reaching the target branch. The anatomical variants of the coronary sinus (CS) are manifold, including tortuosity of a selected branch of the CS, side branch arising at steep angles, smaller diameter, stenotic segments in the side chosen branch, and the competency of luminal valves.^5^

While most anatomical variants are benign, some hold clinical implications, such as venous stenosis for LV lead implantation. Venous valves and tortuosity can be overcome with various techniques; however, stenotic lesion requires dilatation and sometimes stenting to facilitate LV lead implantation.

Venous dilatation has been reported for venous stenosis^4^; however, the risk of venous perforation remains a concern. Venous dissection and perforation have been reported in the past, and they are primarily managed conservatively by abandoning the procedure. A patient with central body perforation of the coronary sinus requires surgery; cases have been reported with patch closure and coronary sinus ligation.^6^ A covered graft stent can be offered in such cases. In our case, we treated the venous perforation with glue injection and completed the procedure. Glues are liquid embolic applied in endovascular embolization for 50 years: the first cyanoacrylate application in peripheral bleeding embolization was described by Dotter in 1975.^7^

The role of glue in the embolization field has grown with the usage of imaging and microcatheters; glues are a consolidated part of the interventional radiology toolbox, especially in its endovascular use. The added advantage of glue in fluoroscopy/cone beam computed tomography-guided procedures is visibility when mixed with Lipiodol.^8^

The concern was LV lead performance in the long term due to glue interaction with LV lead; however, the parameters remained stable for 4 years with excellent clinical outcomes.

## Conclusion

Left ventricular lead positioning remains the most critical determinant predicting CRT response. Anatomical challenges can hinder optimal LV lead placement, particularly venous stenosis. Venous perforation can be a catastrophic complication, managed by glue occlusion and successful LV lead placement.

## Data Availability

The data supporting this study's findings are available from the corresponding author upon reasonable request.

## Lead author biography

Interventional Cardiologist and Cardiac Electrophysiologist, Epic Hospital, Ahmedabad, India, 20 years clinical experience.

## Notes

**Consent:** The authors confirm that written consent for submitting and publishing this case report, including images and associated text, has been obtained from the patient in line with the COPE guidance.

**Funding:** None.
